# Rudimentary Preparation, Compared with Higher Education, for Specialties in Medicine and Surgery*President’s Address, American Academy of Medicine, Baltimore, May 4, 1895.

**Published:** 1895-06

**Authors:** J. McFadden Gaston

**Affiliations:** Atlanta, Ga.


### RUDIMENTARY PREPARATION, COMPARED WITH HIGHER EDUCATION, FOR SPECIALTIES IN MEDICINE AND SURGERY.*

BY J. MCFADDEN GASTON, M.D.,

ATLANTA, GA.

It is taken for granted that an aspirant to the medical profession shall have taken a liberal course of literary study before commencing his professional education. If he has not received the degree of A. B., he should at least have had such mental training as to fit him for the comprehension of instruction in the different departments of medicine. From the standpoint of this academy, the intellectual development which is gained in high-grade schools or colleges is an essential qualification for entering upon the study of medicine. Hence, it is not my purpose to discuss the literary attainments requisite for a medical student.

The attitude of the American Medical College Association and of the Southern Medical College Association has placed the matter of preparatory literary qualification for entering upon the study of medicine upon an entirely satisfactory basis, with the adoption of a uniform standard of literary qualification by all the colleges in the United States.

The only contingency attached to this fundamental condition for entering upon the study of medicine, is the lack of good faith on the part of the medical colleges connected with the college associations in enforcing this requirement for matriculation. There is such an overweening desire of the faculties of some of the medical colleges of this country to add to the number of students in attendance upon their lectures, that they do many things which they should not do, and leave undone many things which they should do, in regard to the preliminary course of study for medical education. We must, however, accept the provision already made, and not reopen the discussion on this point.

Before entering upon the consideration of special medical education, I avail myself of the occasion to congratulate the American

*President's Address, American Academy of Medicine, Baltimore, May 4, 1895.

Academy of Medicine upon the service rendered by this intelligent body in elevating the standard of literary requirement for entering upon the study of medicine. It has not alone been shown that a classical education is essential for the proper comprehension of the technical terms of Latin and Greek origin, but also that the great benefits derived from a liberal education in improving the intellectual faculties, so as to enable the mind of the student to overcome the abstruse points involved in his researches, are of paramount importance. However difficult it may have proved to obtain a knowledge of the so-called ancient languages, and however little some of us may retain of this acquisition after the lapse of years, there is not a fellow of this academy who does not realize that the time and labor spent in securing the degree of A.B. was a good investment of intellectual capital, which is more prized than treasures of pearl and gold.

I shall ask your attention chiefly to the rudimentary and higher technical education for the medical profession. Apart from any consideration of literary qualification for the study of medicine, it is of paramount importance that a preliminary course of private instruction in the rudimentery branches should be received, before a student goes to attend lectures in a medical college. This plan of going over the elementary branches in the office of a private preceptor was the recognized preparation for attending medical lectures fifty years ago in this country. It was not a mere formality to enter upon the study of medicine as an office student of a general practioner at that period.

There were mutual obligations assumed by the student and the preceptor, and daily examinations were made upon the subjects gone over in the text-books. It mattered not how wearied the preceptor might be from his labors of the day, he did not fail to meet the responsibilities of his position as teacher, and the student or students under his care underwent a searching examination each night upon what had been read during the day. The general rule at that time, in the South at least, was for the general practitioner to keep his own drugs and dispense them to his patients; so that his medical students had an opportunity of learning practically how to compound medicines and prepare prescriptions. It was the custom when there were several students in one office to serve alternately as assistants in putting up the medicines under the direction of the preceptor. The most experienced pupils were often placed in charge of those having less familiarity with compounding medicines, so as to secure proper filling of the prescriptions of the preceptor.

It is a notable departure from this *régime* of former times, that while students are under the general supervision of a preceptor at the present day, very few are subjected to the rigid examination upon the text-books before entering college, which is requisite for comprehending the lectures in the several departments taught in the medical schools. The instructions of professors are to all such only as the crackling of thorns under a pot when fire is applied; and they go away from the lecture-room without taking with them any useful knowledge of the subject. Indeed, quite a considerable number of medical students receive no elementary tuition before entering a medical college, and are thus befogged by utter incapacity to grasp the rudimentary elements of medical education. Without learning something of anatomy, biology, and chemistry, the A, B, C, of preparatory work, the student is not fitted to appropriate any of the benefits of a medical lecture given under the supposition of this rudimentary knowledge.

It is not desirable that the student shall take up the higher branches until the lower branches are mastered, because a knowledge of the latter is requisite to a proper comprehension of the former. Over-crowding of the mind of the student with too large a number of topics at one time is also a radical objection to the old *régime* of a uniform curriculum including all the topics included in the course of lectures under the two years plan. The only effective mode of impressing the rudiments upon the mind of the student is to limit the instruction to a few topics at a time; and as the attention is usually directed to a single subject at the outset, under a private preceptor, the best foundation is thus laid for profiting afterwards by the courses of lectures upon elementary matters. When a year has been spent under the faithful tuition of a private preceptor, the pupil enters upon his duties in the college with an appreciation of what is taught, and is prepared to appropriate and digest the intellectual food, as the rations are dealt out by the professor in the classroom from day to day. But it is desirable that digestible food be supplied by the purveyors.

This lack of rudimentary preparation, which was so grave an obstacle to progress in the plan of instruction adopted by the school which had only the two years' course of lectures, is not as patent in the curriculum of those institutions which have entered upon a graded course of three or four years. With a thorough preliminary literary training, a student may accomplish in the first year of a properly graded course nearly all that could be done by private pupilage under the care of a competent preceptor; and yet this general instruction cannot compensate entirely for the special personal teaching of the private office student. In view of the fact that a large proportion of the medical colleges do not insist upon an obligatory compliance with the formal regulation in regard to private office instruction, in the elementary department, before entering upon a course of lectures, it is incumbent that the first year shall be devoted to rudimentary branches. The essential feature of a graded course of three or four years is that the student's attention shall be confined to those subjects first which are preparatory for others. Under the former dispensation of only two courses of stereotyped lectures, which were repeated from year to year from the same manuscript by the professors in our medical colleges, the first course was, to a large extent, lost to the student who had no preparatory instruction. The remarkable feature of such a mode of procedure is that any proficiency in the art of healing the ills to which flesh is heir should ever have been attained; and yet it must be admitted that many entered the medical profession under that *régime*, who, by diligence in study after receiving the degree of M.D., reached distinction as physicians and surgeons. It is held as an axiom, that experience is a dear teacher, and in nothing has this been more strikingly illustrated than in the knowledge of treating diseases obtained by practicing upon the infirmities of mankind. We may well adopt, in this regard, the sentiment that "Man's inhumanity to man makes countless thousands mourn," and pity the poor victims of the doctor's experience.

The fundamental principles underlying medical education cannot be mastered without thorough comprehension of the rudiments, and though there is little to fascinate the medical student in the dry details of the elementary branches, they must be learned before any satisfactory progress can be made in the higher branches. If not well grounded in the rudiments, the superstructure cannot be relied upon to qualify the student for the intelligent exercise of the duties of the physician or surgeon.

It may be thought that drugs are only the tools which are to be used and that practical pharmacy should not occupy the special consideration of the medical student, but without a thorough acquaintance with materia medica the practitioner is at the mercy of every bungling apothecary who may be called upon to fill his prescriptions. The vast array of new preparations, which manufacturers are thrusting upon the medical profession, have supplanted to a large extent the old well-tried medicinal preparations, and unfortunately the glamour of novelty has captured many who are ignorant as to their composition. Experimentation upon an empirical basis is going on throughout this country upon the theories of interested hucksters, who are hawking their wares broadcast amongst the practitioners of medicine. Each manufacturer of drugs is striving to bring out something more attractive than his competitor, and it is most remarkable to note the varied combinations of known remedies, under new names, which are presented to the medical public from year to year, from month to month, from week to week, and even from day to day, by the traveling agents of these establishments. It is evident from the number of samples of these preparations which are left in the offices of practicing physicians, that many are experimenting upon their patients with these articles, and that, like the blind man with a club, they are floundering about without considering whether they may do good or harm to their patients. This is all wrong, and a proper knowledge of pharmacy would save the profession from falling an easy prey to these harpies, and from many a blunder free us, in the application of remedies for the relief of suffering humanity.

A rather ludicrous exemplification of the view taken from the standpoint of the druggist, as to the relative value put upon different articles of the materia medica, occurred in my practice very recently. The time-honored formula of Epsom salts, manna, and infusion of senna leaves was thought to be indicated in the case under treatment, and my prescription went to a druggist, who supposed that he fulfilled his mission by keeping all the new remedies. After looking over the prescription, he handed it back to the bearer, stating that he had no senna leaves in his establishment, as this medicament had gone out of date long ago. Doubtless, he excluded from his shelves, also, such old remedies as ipecac, jalap, castor oil, spirits of turpentine, etc., to make room for the more elegant and fashionable preparations now in the ascendant with most practitioners.

The lesson to be impressed upon the medical student in regard to medication, is that the crude drug embodies all the qualities, which show its effects more completely, in many instances, than the extracts and chemical alkaloids of the same article.

After anatomy, biology, and chemistry are well understood by the student, it is highly important that the materia medica shall be thoroughly comprehended in all its bearings and ramifications. Drugs in their crude state should be recognized by their physical properties and their dosage, independent of their therapeutic applications. The incompatibles, based upon chemical relations, must, of course, be known, as well as the contraindications from their effects upon the organism; and no student can study understandingly the uses of medicines in practice without this knowledge. Much less should any practitioner prescribe those articles of which he does not know the ingredients, as is done so frequently at the present time by men of prominence in the profession of this country.

The plan adopted by the Imperial Academy of Brazil, following, if I mistake not, the French, is to require that the full text of all prescriptions shall accompany every vial or package which is sent out by an apothecary; and while it has some disadvantages in the facility of repetitions, I am fully impressed with its beneficial operations in the great majority of cases, both for the physician and patient.

If this clear record is made by a physician so that his patients may know what they are taking, nostrums and proprietary preparations of unknown components will not figure so conspicuously as at present. The laity would most likely soon become acquainted with the forms of disease in which the family physician used these secret preparations, and be able to dispense with his services in similar cases; so that self-interest would have its influence in preventing their appearance in prescriptions. When the formula accompanies a proprietary preparation the medical profession may employ it without violating any ethical standard, and, in some manner which is inexplicable, these compounds secure the confidence of people more than a regular formula embodied in a physician's prescription. As a rule, the greater the number of ingredients which enter into a remedy the more it is appreciated, on the principle, no doubt, that if one agent does not suit the special disease another may afford relief. Note, for instance, the great reputation of Warburg's tincture, which embodies a vast list of drugs. While it is evident that certain combinations of the materia medica modify in a favorable manner the effect upon the system, the great tendency at the present day to multiply the articles in a prescription should be discouraged, and the student should be taught to rely to a large extent upon the definite effects of distinct medicinal agents. Simplicity in prescriptions is highly commendable, and a proper understanding of pharmacy at the outset of the study of medicine is most conducive to securing this desirable result. It is true that some seem to think that old things have passed away and that all things have become new in the matter of drugs, as in the practice of medicine and surgery. But it should be remembered that the effects of medicines on the human organs are the same to-day that they were in the days of Paracelsus or during any subsequent period of medical observations. We should not then ignore the experience of the past in grasping after the novelties of the present. Hold fast to all the good and eschew all that is bad, is a safe motto.

(*Concluded next month.*)

---

NEVER give stimulants in a case of profuse hemorrhage. The faint feeling or irresistible inclination to lie down, is nature's own method of circumventing the danger, by quieting the circulation and lessening the expulsive force of the heart, thus favoring the formation of a clot at the site of injury.—*Clinique.*

